# Computer-Aided Diagnosis of Micro-Malignant Melanoma Lesions Applying Support Vector Machines

**DOI:** 10.1155/2016/4381972

**Published:** 2016-06-13

**Authors:** Joanna Jaworek-Korjakowska

**Affiliations:** Department of Automatics and Biomedical Engineering, AGH University of Science and Technology, Aleja Mickiewicza 30, 30-059 Krakow, Poland

## Abstract

*Background*. One of the fatal disorders causing death is malignant melanoma, the deadliest form of skin cancer. The aim of the modern dermatology is the early detection of skin cancer, which usually results in reducing the mortality rate and less extensive treatment. This paper presents a study on classification of melanoma in the early stage of development using SVMs as a useful technique for data classification.* Method*. In this paper an automatic algorithm for the classification of melanomas in their early stage, with a diameter under 5 mm, has been presented. The system contains the following steps: image enhancement, lesion segmentation, feature calculation and selection, and classification stage using SVMs.* Results*. The algorithm has been tested on 200 images including 70 melanomas and 130 benign lesions. The SVM classifier achieved sensitivity of 90% and specificity of 96%. The results indicate that the proposed approach captured most of the malignant cases and could provide reliable information for effective skin mole examination.* Conclusions*. Micro-melanomas due to the small size and low advancement of development create enormous difficulties during the diagnosis even for experts. The use of advanced equipment and sophisticated computer systems can help in the early diagnosis of skin lesions.

## 1. Introduction

**Figure 1:**
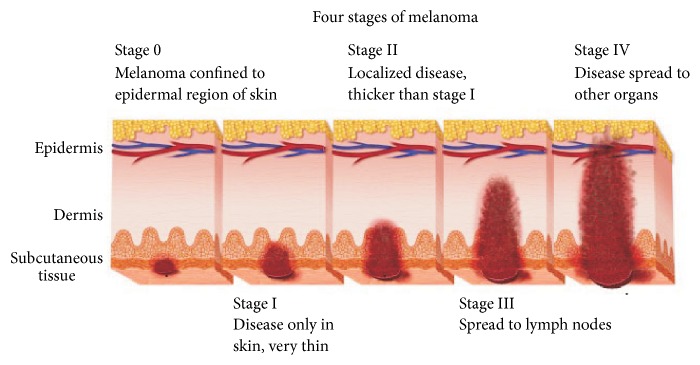
Melanoma is staged in four segments, depending upon the spread of the disease. Stage 0 is also called melanoma in situ: the melanoma cells are only in the outer layer of the skin (the epidermis); Stages I and II mean there are melanoma cells in the layer directly under the epidermis or touching on the next layer down but there is no sign that it has spread to lymph nodes or other parts of the body [11].

Melanoma is a less common but most dangerous form of skin cancer. It starts in the melanocytes or pigment producing cells found in the outer layer of the skin when unrepaired DNA damage to skin cells (most often caused by ultraviolet radiation from sunshine or tanning beds) triggers mutations (genetic defects) that lead the skin cells to multiply rapidly and form malignant tumors (Figure 1). These cells grow out of control and form a tumor. Melanomas are fast-growing and highly malignant tumors often spreading to the nearby lymph nodes, lungs, and brain [1]. Malignant melanoma is likely to become one of the most common malignant tumors in the future, with even a ten times higher incidence rate. Since the early 1970s, malignant melanoma incidence has increased significantly; for example, in the USA it grows approximately by 4 percent every year [1]. The main environmental reason for the rapid growth of melanoma cases is the excessive exposure to ultraviolet (UV) from the sun and sunbeds. Experts estimate about 90% of melanomas are associated with severe UV exposure and sunburns over a lifetime [2].

Due to the high skin cancer incidence, dermatological oncology has become a quickly developing branch of medicine. Nowadays the progress is visible both in primary research concerning pathogenesis of tumors (the role of genes or viruses in tumor development) and in the development of new, more efficient methods of computer-aided diagnosis [1].

This paper is organized in 4 sections as follows.  Section 1 Introduction presents the skin cancer awareness and describes the clinical definition of micro-melanoma skin lesions, motivation of the undertaken research, and related works.  Section 2 Materials and Methods specifies the steps of the designed computer system, including dermoscopy image preprocessing, skin lesion segmentation, feature calculation and selection, and SVM classification method. In Section 3 Results and Discussion the conducted tests and results are described.  Section 4 Conclusions closes the paper and highlights future directions.

*(1) Clinical Importance and Motivation*. The most important parameter which predicts the stage of melanoma is the thickness of the examined lesion (Figure 1). Skin moles with the thickness less than 1 mm are near 100% curable [1]. Seidenari and coworkers reported the direct correlation between diameter and thickness, and as the diameter of in situ melanomas is smaller than that of invasive lesions, it is reasonable to believe that small melanomas are usually in an initial growth phase [3]. Therefore, the aim of each clinician is to detect malignant melanomas when they are still small and thin.

Micro-melanomas represent a minority of diagnosed lesions, their frequency ranging from 1% to 17%. The mean diameter of in situ melanomas is around 1 cm, and invasive melanomas are usually >6 mm [3–6]. In our research we concentrate on micro-melanomas with the diameter lower than 5 mm.

In the light of the above data, prevention and early diagnosis of melanoma become extremely important issues. Micro-melanomas due to the small size and low advancement of development create enormous difficulties during the diagnosis even for experts. Such lesions are so little that they are close to the limit of clinical relevance.

**Figure 2:**
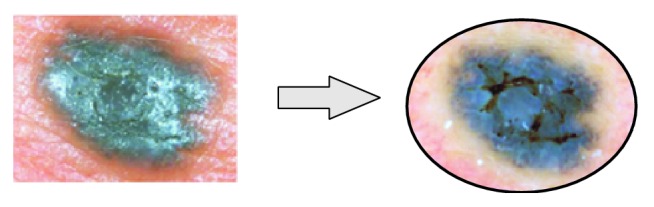
Dermoscopic images of micro-melanoma lesions (based on [1]).

Nowadays, the medical checkup of small moles is possible through a dermoscope (also called digital epiluminescence microscopy or ELM) which consists of a magnifier (typically ×10), a nonpolarised or polarised light source, a transparent plate, and a liquid medium (for nonpolarised light). It enables performing a noninvasive, in vivo examination making subsurface structures of the skin visible (Figure 2).

The dermoscopic diagnosis of pigmented skin lesion is based on the assessment of the presence or absence of different global and local features. Various analytic methods and algorithms have been set forth in the last 25 years. The most important and widely used are pattern analysis, ABCD rule of dermoscopy, 7-point checklist, and Menzies method [1, 7]. The classification of the obtained parameters is the final step of the diagnosis process, where the extracted features are utilized to ascertain whether the lesion is malignant, suspicious, or benign.

*(2) Related Works*. Different groups of researchers have focused on the extraction and classification of significant features to provide distinction between benign and malignant skin lesions; however, when dealing with micro-melanomas only few works have been published but only in the scientific literature of medicine. In 2004 Bono and coworkers described a clinical study of 22 cases of melanoma with the diameter lower than 3 mm. They extracted geometric and dermoscopic features. A lesion was considered suspicious for melanoma when at least one parameter has been positive [8]. In their work they notice that the ABCD diagnostic algorithm becomes redundant while evaluating micro-melanomas and that traditional dermoscopic diagnostic methods often fail. In 2006 Bono et al. published another work about micro-melanoma lesions based on more than 200 medical cases [9]. The clinical evaluation gave a sensitivity of 43% and specificity of 91%. However, using a dermoscope the sensitivity increased to 83% while the specificity dropped to 69%. The described above papers approach the subject only from the clinical point of view.

During patient examinations researchers observe that young inexperienced dermatologists and family physicians have huge difficulties in the correct visual assessment of skin lesions. Due to this obstacle researchers try to implement and build computer-aided diagnosis (CAD) systems for automated diagnosis of melanoma to increase the specificity and sensitivity and make the assessment of the skin mole more simple [10]. Although such computer programs are developed for different diagnostic algorithms, to the best of our knowledge a CAD system to classify micro-melanoma lesions has not been proposed yet. In our approach we propose to divide skin moles into two main groups in terms of the diameter. In this research we concentrate on the evaluation of significant and discriminant features as well as an accurate classification process of only micro-melanoma skin lesions.

## 2. Materials and Methods

After the introduction to the aim of this research we would like to introduce the technical as well as algorithmic solutions. The first part presents the implemented diagnostic system with its particular steps. In the second part we concentrate on the feature extraction and selection as well as the SVM classification method.

**Figure 3:**
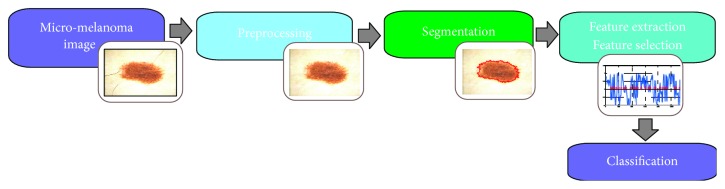
Flowchart of the proposed automated image analysis system.

The implemented application is divided into four stages: preprocessing (image enhancement), skin lesion segmentation, feature extraction and selection, and classification (Figure 3). Since the main aim of this research is to present the feature extraction and classification step, the preprocessing and segmentation stages will be described shortly. A detailed description of these steps can be found in our works [12–14].

### 2.1. Image Enhancement

Dermoscopy images are often deteriorated by noise due to various sources of interference and other phenomena that affect the image acquisition process. Image enhancement techniques are used to refine the analyzed image so that desired image features become easier to perceive and to be detected by the automated image analysis system [15]. Dermoscopic images are inhomogeneous and complex and furthermore they contain extraneous artifacts, such as skin lines, air bubbles, and hairs which appear in almost every image. Therefore, the preprocessing step is essential for dermoscopic images to improve the quality and it is responsible for reducing the amount of artifacts and noise. The preprocessing step includes black frame removal, smoothing of the air bubbles, and black hair inpainting.

The black frame is detected on the basis of the lightness component value in the HSL color space. In order to determine the darkness of a pixel with (*R*; *G*; *B*) coordinates, the lightness component of the HSL color space is calculated [12]:

(1) $$\begin{array}{c} L=\frac{\max\left(R,G,B\right)+\min\left(R,G,B\right)}{2}. \end{array}$$

A pixel is considered to be black when the lightness value is less than 15 (range of the lightness value [0 : 255]). For the smoothing of air bubbles and light hairs we use the Gaussian filter.

**Figure 4:**
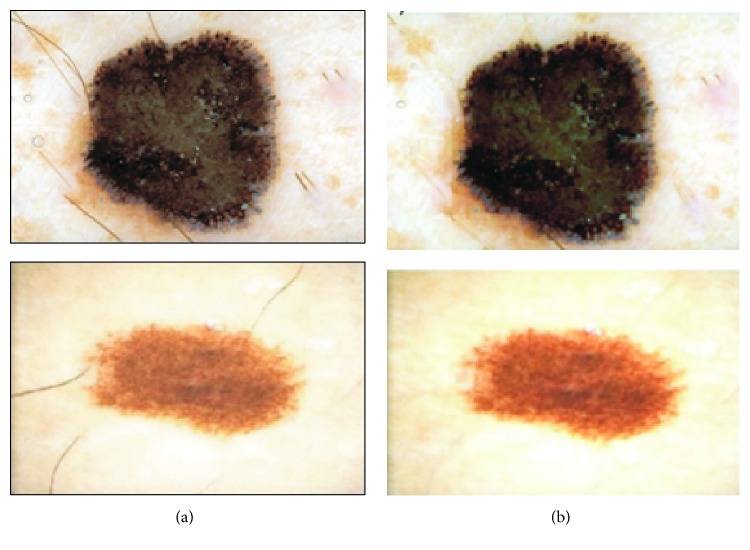
Outcome of the preprocessing step: (a) dermoscopic input image and (b) image after black frame removal and hair inpainting.

The removing of thick black hair is the most important step in dermoscopy image preprocessing since it influences the segmentation and feature extraction step. Firstly, the dermoscopic RGB image is being converted into grayscale with the NTSC 1953 standard. Secondly, the black top-hat transform, which is a morphological image processing technique, is used to detect thick, dark hairs. The result of this step is the difference between the closing operation and the input image:

(2) $$\begin{array}{c} T_{w}\left(\;I\;\right)=I\circ b-I, \end{array}$$

where ∘ denotes the closing operation, *I* is the grayscale input image, and *b* is a grayscale structuring element [15]. The last step is the hair inpainting. Hair line pixels are replaced with values calculated on the basis of the neighborhood pixels (Figure 4).

### 2.2. Segmentation Algorithm

**Figure 5:**
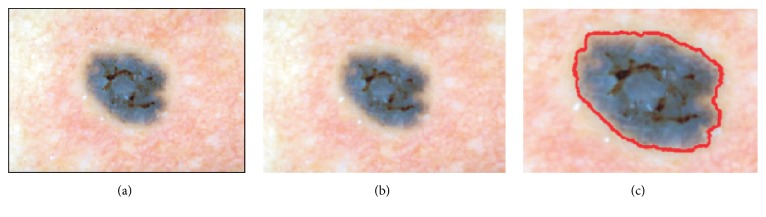
Dermoscopic image segmentation using region-growing method: (a) dermoscopic input image, (b) image after enhancement, and (c) segmented skin mole.

The principal goal of the segmentation process is to partition an image into regions that are homogeneous in terms of pixel intensity or specific features [15]. In medical imaging, segmentation is important and crucial for feature extraction and image measurements and classification. Precision of classification of a tumor depends greatly on the accuracy of the extracted tumor area, as most of the features are calculated on this basis. During our work we have compared different segmentation methods [13, 14]. Based on the previous research the applied segmentation algorithm for the skin lesion extraction is based on seeded region-growing algorithm. The region-growing algorithm, also called region merging, looks for groups of pixels that have similar intensities, whereas thresholding methods focus on the difference of pixel intensities [15]. The main advantage of the region-growing algorithm is that it is capable of correctly segmenting regions that have the same properties and are spatially separated. Because the healthy skin in dermoscopy images is mostly homogenous the region-growing algorithm can be applied to detect it. For the skin lesion segmentation we select one seed which is located in the left corner, on the healthy skin. It gives us the certainty that we will separate the homogeneous background from the skin mole. The region is iteratively grown by comparing all unallocated neighboring pixels to the region. The region-growing process consists of picking a seed from the set, investigating all 4-connected neighbors of this seed, and merging suitable neighbors to the seed. The seed is then removed and all merged neighbors are added to the seed set. The region-growing process continues until the seed set is empty.  Figure 5 presents the results of preprocessing and segmentation steps for micro-melanoma skin lesion.

### 2.3. Dermoscopic Image Analysis

After the preprocessing and segmentation steps, the dermoscopy image is prepared for a detailed analysis. Now, it is necessary to analyze the image and recognize critical parameters that can indicate melanoma. These properties are basically the same ones that a physician looks for. In this research we concentrate on two of the most informative visual cues in dermoscopy image interpretation including shape and texture. Shape parameters correspond with the diagnostic algorithm called the ABCD rule of dermoscopy, while the texture parameters correspond with the diagnostic algorithm 7-point checklist.

#### 2.3.1. Feature Extraction

Many different image features have been proposed for the analysis of medical images including intensity, geometrical, morphological, fractal dimension based, and texture features. The feature extraction step can be considered as data compression that removes additional and irrelevant information while preserving relevant information in a data vector. Features selected for the calculation of detected skin moles are based on the knowledge and experience about the properties and differences in appearance between the benign and malignant skin lesions. However, to find out the most redundant and effective features, we extract a large number of initial features. The proposed features are identifiable, translatable, rotation and scale invariant, noise resistant, and reliable.

*Shape Features (Geometrical Features)*. Shape-based image retrieval consists of measuring of similarity between shapes represented by their features. Shapes that have large differences can be identified with the geometrical features; however even small changes can affect the final value of the feature. We calculated parameters including major and minor axes length, eccentricity (measure of aspect ratio: it is the ratio of the major axis to the minor axis), elongation (based on the bounding box), compactness (normalized version by Haralick), ellipse variance, rectangularity (ratio area of shape and area of the minimum bounding box), convexity (ratio of perimeter of convex hull and contour), and solidity (ratio of area of convex hull and shape area). 

*Asymmetry*. Asymmetry is the major parameter in the diagnostic algorithm ABCD rule of dermoscopy. We quantify asymmetry calculating the symmetry distance (SD) proposed by NG in 1997 [16, 17]. It is the calculated result of typical displacement among the number of vertices when the skin lesion shape is transformed into its symmetric shape [18]. The SD value is measured as

(3) $$\begin{array}{c} SD=\frac{1}{n}\overset{n-1}{\underset{i=0}{\sum }}\left\|\;Pi-\hat{P}i\;\right\|. \end{array}$$

*Color Variegation*. A skin lesion may have a variety of colors including tan, dark brown, black, blue, red, and, occasionally, light gray. Melanomas are usually characterized by three or more colors and in about 40% of melanomas even five or six colors are present. We analyze the color variation through four different channels including red, green, blue, and intensity. The intensity channel for each pixel *I*(*x*, *y*) is defined:

(4) $$\begin{array}{c} I\left(\;x,y\;\right)=\sqrt{R^{2}\left(x,y\right)+G^{2}\left(x,y\right)+B^{2}\left(x,y\right)}. \end{array}$$

The following parameters are calculated for assessing the color variegation [7]:

(5) cv1=log⁡σRμRcv2=log⁡σGμGcv3=log⁡σBμBcv4=log⁡σIμI,

where *μ* is the mean value and *σ* denotes standard deviation.

*Texture Features*. Examination of dermoscopy images often requires the interpretation of global pattern appearance, which can be generally described with terms such as regularity, homogeneity, smoothness, or grain. These attributes are related to the local intensity variations and can be quantified by using texture metrics. For the analysis of texture parameters we use cooccurrence matrix measures that are computed from a 2D histogram, which preserves spatial information.

A cooccurrence matrix is a matrix that represents the distribution of cooccurring values at a given offset. A cooccurrence matrix *C* is defined over an *n* × *m* image *I*, parameterized by an offset (Δ*x*, Δ*y*), as [7]

(6) CΔx,Δyi,j=∑p=1n ∑q=1m1,if  Ip,q=i,  Ip+Δx,q+Δy=j0,otherwise.

The values *i* and *j* and size of *C* are determined by the values in *I*.

The texture analysis for dermoscopic images is performed for 8-bit grayscale images, quantized to 256 values, and is referred to as gray-level cooccurrence matrices (GLCM).

Textural features are frequently used in advanced melanocytic lesion analysis [7]. Celebi et al. suggested uniformly quantizing the dermoscopy image to 64 gray levels and then extracting 8 Haralick features for each one of the four orientations using single pixel offsets [7, 19–21]. The following parameters have been calculated:

(7) maximum  probability=maxi,j⁡ ci,j,energy=∑i∑jci,j2,entropy=−∑i∑jpi,jlog⁡ci,j,dissimilarity=i−jci,j,contrast=∑n=0N−1∑i∑jci,j ∣ i−j=n,inverse  difference=∑ci,j1+i−j,inverse  difference  moment=∑ci,j1+i−j2,correlation=ijpi,j−μxμyσxσy,

where *c*(*i*, *j*) is the value of the normalized cooccurrence matrix at indexes (*i*, *j*), *N* is the number of gray levels, *μ*_*x*_ and *μ*_*y*_ are the mean values of the rows and columns of *c*, *σ*_*x*_ and *σ*_*y*_ are the respective standard deviations, and the symbol ∣ indicates a condition that must be valid.

Since the range of values of raw data varies widely, the feature scaling step has to be done before feature selection and classification. Feature scaling is a method used to standardize the range of independent variables or features of data. In this work we rescale the range of features to scale the range in [−1,1]. The formula is given as

(8) $$\begin{array}{c} x^{\prime }=2\cdot \frac{x-\min\left(x\right)}{\max\left(x\right)-\min\left(x\right)}-1. \end{array}$$

#### 2.3.2. Feature Selection

In the next step we select the most suitable features from the set of all features described in the previous section. There are many criteria to describe effective features, but they should possess the following advantages: large interclass mean distance, small interclass variance, insensitivity to extraneous variables, and low correlation with other features [15].

In this work we propose to use the correlation-based feature selection (CFS) algorithm. CFS relies on a heuristic for evaluating the worth or merit of a subset of features. This heuristic takes into account the usefulness of individual features for predicting the class label along with the level of intercorrelation among them.

If the correlation between each of the components in a test and the outside variable is known and the intercorrelation between each pair of components is given, then the correlation between a composite consisting of the summed components and the outside variable can be predicted from [22]:

(9) $$\begin{array}{c} r_{zc}=\frac{k\bar{r_{zi}}}{\sqrt{k+k-\left(k-1\right)\bar{r_{ii}}}}, \end{array}$$

where *r*_*zc*_ is correlation between the summed components and the outside variable, *k* is number of components (features), $\bar{r_{zi}}$ is average of the correlations between the components and the outside variable, and $\bar{r_{ii}}$ is average intercorrelation between components.

Equation (9) shows that the correlation between a composite and an outside variable is a function of the number of component variables in the composite and the magnitude of the intercorrelations among them, together with the magnitude of the correlations between the components and the outside variable [22].

The following features have been chosen for the classification process: compactness, solidity, symmetry distance, color variegation features, variance of entropy, contrast, dissimilarity, and ellipse variance.

### 2.4. Classification Using Support Vector Machine (SVM)

After the set of discriminative features has been selected, a classifier can be designed for the evaluation of micro skin moles. The Support Vector Machine (SVM) is a supervised learning model used for data analysis, pattern recognition, classification, and regression analysis. An SVM training algorithm builds a model that assigns new samples to a related class. An SVM model is a representation of samples as points in space; a linear function is used so that the examples of the separate classes are divided by a clear gap.

Given a training set of instance-label pairs (**x**_*i*_, **y**_*i*_), *i* = 1,…, *l*, where **x**_*i*_ ∈ *R* ^*n*^ and **y** ∈ {1, −1}^*l*^, the SVM requires the solution of the following optimization problem [23]:

(10) minw,b,ξ 12wTw+C∑i=1lξisubject  to yiwTϕxi+b≥1−ξi ξi≥0,

where training vectors **x**_*i*_ are mapped into a higher dimensional space by the function *ϕ*. The SVM finds a linear separating hyperplane with the maximal margin in this higher dimensional space. *C* > 0 is the penalty parameter of the error term.

Moreover, *K*(**x**_*i*_, **x**_*j*_) ≡ *ϕ*(**x**_*i*_)^*T*^ *ϕ*(**x**_*j*_) is called the kernel function [23]. We use the commonly implemented Radial Basis Function (RBF) defined as

(11) $$\begin{array}{c} K\left(\;x_{i},x_{j}\;\right)=\exp\left(\;-\gamma {\left\|x_{i}-\right.\left.x_{j}\right\|}^{2}\;\right),\;\gamma >0. \end{array}$$

### 2.5. Model Evaluation Method

For the evaluation of the designed algorithm we use cross-validation which is a technique for assessing how the results of a statistical analysis will generalize to an independent dataset. Cross-validation (CV) is primarily a way of measuring the predictive performance of a statistical model. In this approach, the so called *k*-fold cross-validation method has been used. In *k*-fold cross-validation, the original sample is randomly split into *k* smaller sets. The following steps are undertaken: a model is trained using *k* − 1 of the folds as training data and the resulting model is validated on the remaining part of the data.

The performance measure reported by *k*-fold cross-validation is then the average of the values computed in the loop.

## 3. Results and Discussion

### 3.1. Database Specification

The described algorithm for the automatic classification of micro-melanocytic lesions has been tested on dermoscopic images from a widely used Interactive Atlas of Dermoscopy [1] and a private database. Images for this atlas have been provided by two university hospitals (University of Naples, Italy, and University of Graz, Austria) and stored on a CD-ROM in a JPEG format. The documentation of each dermoscopic image was performed using a Dermaphot apparatus (Heine, Optotechnik, Herrsching, Germany) and a photo camera (Nikon F3) mounted on a stereomicroscope (Wild M650, Heerbrugg AG, Switzerland) in order to produce digitized ELM images of skin lesions. All of the images have been assessed manually by a dermoscopic expert with extensive clinical experience.

Furthermore, all of the descriptions of skin cases were based on histopathological examination of the biopsy material. In order to develop and test the automatic procedure for the classification of micro-melanocytic skin lesions, 200 images with different resolutions, ranging from 0.033 to 0.5 mm/pixel, were chosen. The database included 70 malignant melanomas and 130 benign cases.

The preprocessing step (black frame removal and hair removal) as well as the segmentation step (border error less than 6%) did not affect the further research [13, 14].

### 3.2. Statistical Analysis

Diagnostic exactness is often used to mean the fraction of cases on which the automated image analysis system gives a correct answer, where correctness is determined by comparing the diagnostic decision to some definition of “truth” [15]. There are different ways to define the “truth.” In our case, we depend on the separate gold standard which is determined by the results of biopsy for the dermoscopic images.

The way to assess the accuracy of the designed classification system is to compare its results against the gold standard. The analyzed task is a binary problem, where the answer can be “1” when the lesion is malignant or “0” when it is benign. To measure the diagnostic performance we use a pair of statistics parameters including sensitivity and specificity, which are not affected by the disease prevalence.

The sensitivity or True Positive rate is the probability that the lesion is said to be malignant given that it is. This can be estimated by relative frequency [15]:

(12) $$\begin{array}{c} sensitivity=\frac{\#TP}{\#TP+\#FN}, \end{array}$$

where TP is the True Positive answer and FP is the False Positive answer.

On the other hand, we calculate specificity (False Positive rate) which is defined as the proportion of the True Negatives against all negative results. Specificity is defined by the following equation:

(13) $$\begin{array}{c} specificity=\frac{\#TN}{\#TN+\#FN}, \end{array}$$

where TN is the True Negative answer and FN is the False Negative answer.

Table 1 shows the classification results obtained with the implemented algorithm in a confusion matrix.

**Figure 6:**
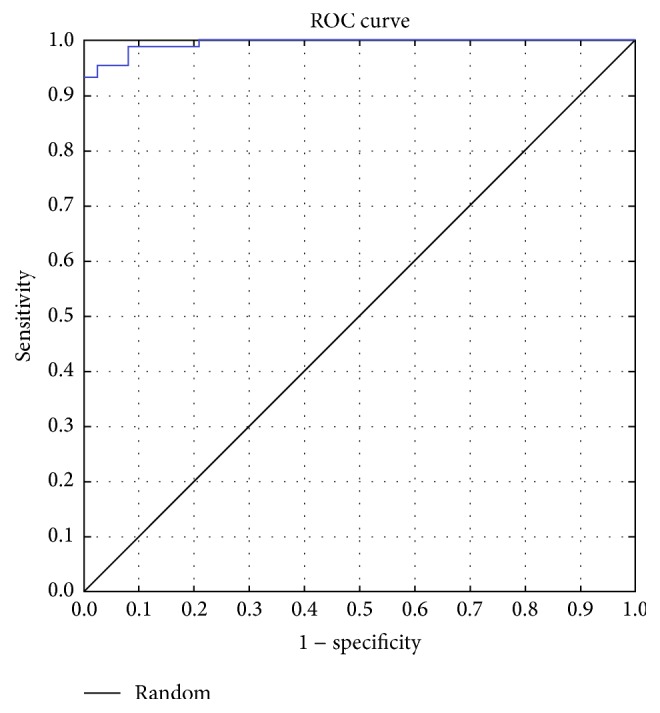
ROC plot for the SVM classifier (AUC = 93.24%).

Another dominant technique for assessing computer classification performance is the receiver operation characteristic (ROC) analysis. A ROC curve is a plot of sensitivity versus 1 − specificity (Figure 6). Area under the ROC curve is the most commonly used accuracy index. The area under a perfect-accuracy ROC curve, when sensitivity and specificity reach 1.0, is also 1.0. Another interesting fact is, that for random guessing is the positive diagonal line and the area under the curve is 0.5 [15].

The conducted results (sensitivity = 90%, specificity = 96%, and AUC = 93.24%) achieved by the implemented micro-melanoma classification system are much better than the results described in [8, 9]. Our method allowed classification of micro-melanomas very precisely and we can confirm that the classification of micro skin lesions has to be done separately from the classification of developed skin moles. Overall, our experiments clearly show that the classification of micro skin moles, which causes problems, can be supported by a powerful system based on proposed artificial neural network.

## 4. Conclusions and Future Plans

Due to the difficulty and subjectivity of human interpretation, the computerized image analysis techniques have become an important tool in the interpretation of dermoscopic images. In this research we propose a new approach for the classification of melanocytic skin moles. The results obtained within this study indicate that the proposed algorithm can be used for the diagnosis of very small skin moles. These conclusions were reached after comparing computer classification with biopsy outcome using statistical measurements and ROC curve. The automatic procedures have been tested and it seems to be a very promising software tool supporting the physician. The proposed diagnostic system can be used not only by the young, inexperienced dermatologist but also, first and foremost, by family physicians. This can be a great opportunity for people that live in remote and rural areas outside the regional centre and frequently find it difficult to make an appointment with a dermatologist. The importance of diagnosing a melanoma in the early stage and the reduction of the melanoma-related mortality rate can be achieved by precise computer-aided diagnostic systems.

However, computer classification of micro-melanomas must also be further developed and tested on a larger dataset before it can be used clinically and before its potential in reducing the mortality rate of malignant melanoma can be realized in clinical medicine.

## Figures and Tables

**Table 1:** List of parameters evaluated by the proposed method.

Desired result	Output result		Total
	Benign	Malignant	
Benign	65	5	70
Malignant	7	123	130
	*Sensitivity 90%*	*Specificity 96%*	200
